# Integrin α1 promotes tumorigenicity and progressive capacity of colorectal cancer

**DOI:** 10.7150/ijbs.37275

**Published:** 2020-01-16

**Authors:** Hai Li, Yong Wang, Shi-kuo Rong, Ling Li, Tuo Chen, Ya-yun Fan, Yu-feng Wang, Chun-rong Yang, Chun Yang, William C. Cho, Jiali Yang

**Affiliations:** 1 Department of Colorectal Surgery, General Hospital of Ningxia Medical University, Yinchuan, Ningxia 750004, China.; 2 College of Clinical Medicine, Ningxia Medical University, Yinchuan, Ningxia 750004, China.; 3 Department of Orthopedics, Shangluo International Medical Center Hospital, Shangluo, Shanxi 726000, China.; 4 Department of Occupational and Environmental Health, Public Health and Management School, Ningxia Medical University, Yinchuan, Ningxia 750004, China.; 5 Department of Gynaecology, Jingzhou Hospital Affiliated to Tongji Medical College, Huazhong University of Science and Technology, Jingzhou, Hubei 434000, China.; 6 Department of Gastroenterology, Hospital of Chengdu University of Traditional Chinese Medicine, Sichuan 610072, China.; 7 Department of Clinical Oncology, Queen Elizabeth Hospital, Kowloon, Hong Kong.; 8 Key Laboratory of Ministry of Education for Conservation and Utilization of Special Biological Resources in the Western, and College of Life Science, Ningxia University, Yinchuan, Ningxia 750021, China.

**Keywords:** Integrin α1 (ITGA1), colorectal cancer, progression, tumorigenicity

## Abstract

Colorectal cancer (CRC) is the second leading cause of death globally. Integrin α1 (ITGA1) belongs to integrin family and involves in regulating cell adhesion, invasion, proliferation and tumorigenicity, its expression is up-regulated in various cancers, including CRC. However, the molecular understanding and clinical relevance of ITGA1 in the development and progression of CRC remain unclear. In the present study, we detected ITGA1 in 50 CRC tissues and adjacent non-cancerous tissues, sera from 100 CRC patients and 50 healthy subjects, and four CRC cell lines using immunohistochemistry staining, enzyme-linked immunosorbent assay and Western blotting. We found that the ITGA1 protein was significantly higher in human CRC tissues and cell lines than both paired non-tumor tissues and normal cells, respectively. In addition, the serum concentration of ITGA1 was also higher in CRC patients compared to the healthy subjects (p<0.01) and was significantly associated with metastatic TNM stages (p<0.0001) and circulating carbohydrate antigen 199 (CA199) (p<0.022). Furthermore, down-regulation of ITGA1 with transfecting LV-shITGA1 inhibited the progressive capacity of cell migration and invasion in CRC SW480 cell line and the tumorgenicity in nude mice. In functional studies, ITGA1 knockdown also inhibited Ras/ERK signaling pathway by decreasing the expression of Ras, p-Erk1/2 and c-Myc in SW480. Contrastly, when evelated expression of ITGA1 in NCM460 coincided with the increased expression of Ras, p-Erk1/2 and c-Myc. Taken together, our findings suggest that *ITGA1* is an oncogene with a capability to promote CRC cell migration, invasion and tumorigenicity by activating the Ras/Erk signaling, implying that it may be a novel target for the diagnosis and treatment of CRC, and warrants further investigation.

## Introduction

Colorectal cancer (CRC) is the most common gastrointestinal cancer. The incidence and mortality rate of CRC are rising worldwide 1, 2. Early diagnosis and detection of CRC can reduce patient mortality 3, 4, but the median survival rate of patients with advanced CRC is still low 1. It is well known that carcinoembryonic antigen (CEA) and carbohydrate antigen 199 (CA199) are popular tumor biomarkers for the CRC, however, studies shown that both serum CEA and CA199 have been showed unsatisfactory performance in CRC screening 5, 6, both of them are recommended as circulating tumor markers in CRC for tumor detecting and monitoring responses to therapy according to the European Society for Medical Oncology (ESMO) and Chinese Society of Clinical Oncology (CSCO) clinical practice guidelines for CRC diagnosis 7, 8. Therefore, it is necessary to identify new biomarker for early diagnosis and therapeutic target for CRC patients.

Integrins are a large family of transmembrane glycoproteins and heterodimers of noncovalently associated α and β subunits. To date, there are 18α and 8β subunits have been identified that can form 24 distinct protein receptors with binding to the corresponding ligands to mediate adhesion between cells and basal membrane of cells 9-11. In this regard, integrins have gained great interest in studies of tumorigenesis, progression and metastasis, owing to their abilities to control the adhesion, migration, proliferation, invasion, and tumorigenicity of cancer cells 11, 12. Among them, the integrin α1 (ITGA1) is one of the important members of integrins 13, which was reported to significantly up-regulate in colorectal tumors and surrounding the tumour cells by immunohistochemical analysis 12, 14. Boudjadi et al demonstrated that integrin α1β1 was positively correlated with CRC cell invasion and proliferation by activating the focal adhesion kinase (FAK)-src/p130Cas-JNK signaling cascades 6, 12, 14, 15 and the Ras/MEK/ERK signaling pathway 16. However, the role and underlying mechanism of ITGA1in the colorectal adenoma is still not clear.

Intriguingly, our recent mRNA expression profiles of human colorectal tissues and precancerous detecting with the Affymetrix Human Genome U133 Plus 2.0 Array shown that *ITGA1* was one of the most up-regulated genes (p=1.95E-17) in human cancer tissues compared with the corresponding precancerous tissues. In this study, we investigated the roles of ITGA1 in CRC, by detecting its expression in CRC tissues, serum and cancer cell lines, evaluating the correlation of ITGA1 with pathological features and circulating CA199, analyzing the biology of ITGA1 in CRC by regulating the Ras/Erk signaling pathway, and examining its effects on the progressive properties and tumorigenicity in CRC cells.

## Materials and methods

### Ethics statement

The protocols for using human CRC tissues and serum in this study were approved by the Ethics Committee for the Conduct of Human Research at General Hospital of Ningxia Medical University (GHNXMU-H-2012-0121). Written consent was obtained from each participated patients. They were over 18 years of age and given informed consent before specimen were collected. All experiments using animals were performed in accordance with the guidelines of Chinese Council on Animal Care and approved by the Committee for Animal Care and Use of Ningxia Medical University (NXMU_CACU 2014-2017).

### Human CRC tissue samples, serum and cell lines

50 CRC with paired adjacent non-tumor tissues (at least 5 cm apart from tumors) and 100 tumor serum samples were collected from CRC patients who underwent an operation in Department of Colorectal Surgery, Ningxia Medical University General Hospital. The pathologic tumor staging was followed according to the International Union Against Cancer (2009). Surgically resected samples were taken from patients that had not received prior radiotherapy, chemotherapy or immunotherapy. 50 serum samples of healthy subjects were also collected from individuals who underwent a routine physical examination in the hospital. The normal colonic epithelial cell line NCM460, human colon cancer cells HCT116, HT29 (*in situ* colon cancer cells), SW620 (lymph node metastatic colon cancer cells), SW480 (*in situ* cancer cells) were purchased from American Type Culture Collection (ATCC, Mannasas, VA, USA) and were cultured in RPMI-1640 (GIBCO-Invitrogen, Carlsbad, CA, USA) supplemented with 10% fetal bovine serum (FBS) (Invitrogen), 1% penicillin/streptomycin at 37 ^º^C in a humidified atmosphere of 5 % CO_2_.

### Immunoblotting analysis

Total proteins lysates of tissue or whole cell extract were prepared in a lysis buffer (Nanjing, KeyGen Biotech. Co. Ltd, China) plus protease and phosphatase inhibitor for 1 hr on ice. And then centrifuged at 12,000 xg for 10 mins at 4 ^º^C, and collected the supernatants as crude cell extracts. Each protein (50 μg) was resolved and separated by a 10% sodium dodecyl sulfate-polyacrylamide gel (SDS-PAGE) using the Bio-Rad standard electrophoresis for electrophoresis about 1-2 h. Then the protein was transferred onto a polyvinylidene fluoride (PVDF) membrane (Millipore, Burlington, MA), which was activated for 3 mins by methyl alcohol before used. After blocked with 5% non-fat milk powder in Tris-buffered saline (TBS) for 1 hr at room temperature (RT), the membrane was incubated with rabbit anti-ITGA1 (1: 500, Proteintech, New Jersey, USA), ERK, p-ERK, RAS and c-Myc. All these four antibodies were diluted at 1:1,000 with the same supplier, Cell Signaling Technology, USA as well as anti-β-actin (1:5,000, Proteintech, New Jersey, USA) for overnight at 4 ºC. The membranes were washed with 1×TBST (0.1% Tween-20 in TBS) three times, subsequently incubated with horseradish peroxidase (HRP)-conjugated goat anti-rabbit IgG (ZSGB-Bio Origene, Beijing, China) (1:1,000 in blocking buffer) for 1.5 hr at RT. The chemoluminescence signals were visualized using the enhanced chemiluminescence (ECL) reagent (Advansta, Menlo Park, CA, USA). The protein expression level was semi-quantified using Image lab (Bio-Rad) software. The relative ratio of the net intensity of each sample normalized by the β-actin internal control was calculated as densitometric arbitrary units (A.U.) which served as an index of relative expression of a protein of interest 17, 18.

### Detection of ITGA1 by Enzyme-Linked Immunosorbent Assay (ELISA)

The concentration of ITGA1 protein in sera was measured by ELISA kit (Cusabio Inc, Wuhan, China) accordingly to the manufacturer's instructions. Briefly, all serum specimens were diluted 10 times with the standard dilution. Diluted samples were added 100 μL to each well and incubated at RT for 1 h. Then the wells were washed with a high ionic strength buffer and added horseradish peroxidase (HRP)-conjugated anti-human IgG supplied with the kit for 1 hr at RT. The wells were extensively washed for three times and 100 μL trimethylbenzene solution was added for 30 mins at RT. 100 μL stopping solution was added to each well before the optical density was measured at 450 nm. The absorbance values (OD_450_ nm) of the corresponding specimens were obtained and then converted into a concentration (ng/mL) through standard curve using Curve Expert 1.4 Software. Data represented the mean ± SD at least three independent triplicated experiments.

### Immunohistochemistry staining

For immunohistochemistry (IHC) staining, paraffin sections (5 μm in thickness) of the clinical human colon cancer tissues, adenocarcinoma and the paired tumor-adjacent tissues and tumor tissues of nude mice were deparaffinized, rehydrated through graded alcohol solution, and then boiled (100±5 ^º^C) in 10 mM sodium citrate pH 6.0 for 3 mins in an autoclave and then cooled down to RT for antigen retrieval. In order to eliminate endogenous peroxidase activity, tissue sections treating with 3% hydrogen peroxide at RT for 20 mins. Then the sections were blocked with blocking buffer (5% sheep serum in PBS) for 2 hrs at RT to block the non-specific binding. Paralleled sections were incubated with rabbit anti-human ITGA1 antibody (1:200, ab154845, Abcam, Cambridge, UK) and rabbit anti-human Ki67 antibody (1:200, 60065-1-Ig, Proteintech Group, Rosemont, IL, USA) overnight at 4 °C. After washing for 3 × 5 mins in PBS, sections were incubated with peroxidase labeled donkey anti-rabbit IgG (ZSGB-Bio Origene, Beijing, China) (1:500 in blocking buffer) for 30 mins at RT. After washing for 3 × 5 mins in PBS, the ITGA1 and Ki67 signal was developed in 3, 3'-diaminobenzidine (DAB) peroxidase substrate kit (Vector Laboratories, Burlingame, CA, USA) and subsequently counterstained with hematoxylin. The images of stained sections were taken using a high-powered upright microscope (Leica DM3000, Germany). The mean of gray-level density and numbers of positive-ITGA1 and Ki67 cells were analyzed using Image J (https://imagej.nih.gov/ij/). The IHC-stained sections were then evaluated by three experienced pathologists independently in a blind manner. The median value of the H-score was chosen as the cutoff to dichotomize into high and low expression subgroup.

### Generation of lentiviral vectors and cell lines overexpressing and silencing *ITGA1* gene

We found that ITGA1 expression was higher in certain CRC cell lines (SW480, SW620, HT29, HCT116), especially in highly metastatic SW480 cells than in normal colonic epithelial NCM460 cells. Therefore, both a relatively high or low endogenous expression of ITGA1 in SW480 and NCM460 respectively were selected for further study. In order to overexpress the endogenous ITGA1 in NCM460 cells, a lentiviral vector expressing ITGA1 was generated by Shanghai GeneMarker Inc (Shanghai, China). Full-length cDNA encoding human ITGA1 (NM_181501) protein was cloned into the downstream GV358 lentiviral vector using AgeI/AgeI sites, and the resultant viral vectors were designated as LV-ITGA1. The vector contained a SV40 promoter-derived Puromycin selective marker. In order to silent endogenous ITGA1 in SW480 cells, lentiviral vector expressing shRNA to ITGA1 gene was also generated by targeting *ITGA1* mRNA (target: NM_181501) (GenBank ID) from GeneMarker Inc (Shanghai, China), were: Seq 1 (for SW480-ITGA1-sh1 cells), 5'-GGCCAACCCAAAAACAGAAC-3'; Seq 2 (for SW480-ITGA1-sh2 cells), 5'-GGTAGATCCAACTTTACACA-3'; Seq 3 (for SW480-ITGA1-sh3 cells), 5'-TATCCAAACATGTCTTCCAC-3'. Lentiviral vectors of scramble RNA and no transgene empty lentiviral vector (LV-NC) were also generated for the respective infection controls of shRNA-mediated knockdown and overexpression. For generation of stable cell lines, the cells (30-40% confluency) were selective with 2.5 μg/mL puromycin for one or two weeks.

### Cell scratch assay

The cell scratch assay is widely used to study the coordinated cell movement; we employed the cell scratch assay to evaluate the CRC cell migration capacity. The lentiviral infected NCM460 and SW480 were seeded into 80% confluent in 6-well plates and scratched with 200 μL pipette tips, and then used pre-warmed PBS to wash resultant unattached cells. After 48 hrs, the wounded monolayers were stained with 0.1% crystal violet solution. The images of wounded areas was taken under an inverted microscope (Leica, Wetzlar, Germany) and photographed. The distance of closure was quantified with the Image-Pro plus processing program. Migration index (the ratio of wound healing area to scratch area) was used to evaluate the cell migration capacity 17.

### Transwell assay

The filters (8 μm pore; Coster, Cambridge, MA) were coated with 100 μL Matrigel (BD Bioscience, San Jose, CA, USA), which was diluted in 1:2 with serum-free 1640 medium, and incubated at 37^º^C in 5% CO_2_ for 30 mins for Matrigel 2×10^4^ lentivirally infected CRC cells in 100 μL of culture medium were seeded in the upper Matrigel chamber and a 700 μL 0.2% FBS conditional medium was added in the lower chamber. Incubated at 37^º^C for 12 hrs, then removed and wash twice by PBS. After incubation at 37^º^C for 12 hrs, the cells passed though the filters were washed twice by PBS and fixed with 4% of paraformaldehyde for 20 mins and subsequently stained with 1% crystal violet for 20 mins. The number of cells was counted under the microscope. At least three independent experiments were performed in each group.

### Tumorigenesis and treatment with expression or silence ITGA1 in CRC cells* in vivo*

6-8 week-old female nude mice (Beijing Vital River Lab Animal Technology Co., Ltd., Beijing, China) were feeded in the specific pathogen-free (SPF) environment and used to evaluate the tumorigenic capacity of CRC cells. Forthe tumorigenesis test, NCM460 cells and SW480 were infected with lentiviral vector of ITGA1 or ITGA1 shRNA, respectively. The cells were then selectively screened in medium containing puromycin for one week; prior to being subcutaneously (s.c.) transplanted in nude mice with 200 μL Matrigel containing 2×10^6^ virus-transduced CRC cells. After 3 weeks following the cell transplantation, the animals were euthanized and collected for evaluation of tumorigenicity. The diameter of tumor was measured. And then the tumor tissues were fixed in 10% neutral formalin and subsequently embedded in paraffin for further WB ananlysis for ITGA1 expression and IHC staining with cell proliferative marker Ki67.

### Statistical analysis

All data in this study were presented as the mean ± standard deviation (SD) of at least three independent experiments for each condition. All IHC images were quantified by intensity of density (IOD) using Pro Plus 6 (Media Cybernetics Inc.). All statistical analysis was performed using PRISM 6 (GraphPad software, La Jolla, CA, USA). Both one-way ANOVA and *t*-test were used for statistical evaluation. Significant differences were assigned to p values <0.05, <0.01 and <0.001 denoted by *, ** and ***, respectively.

## Results

### Elevated ITGA1 protein in tumor tissues and sera of CRC patients

**Figure 1:**
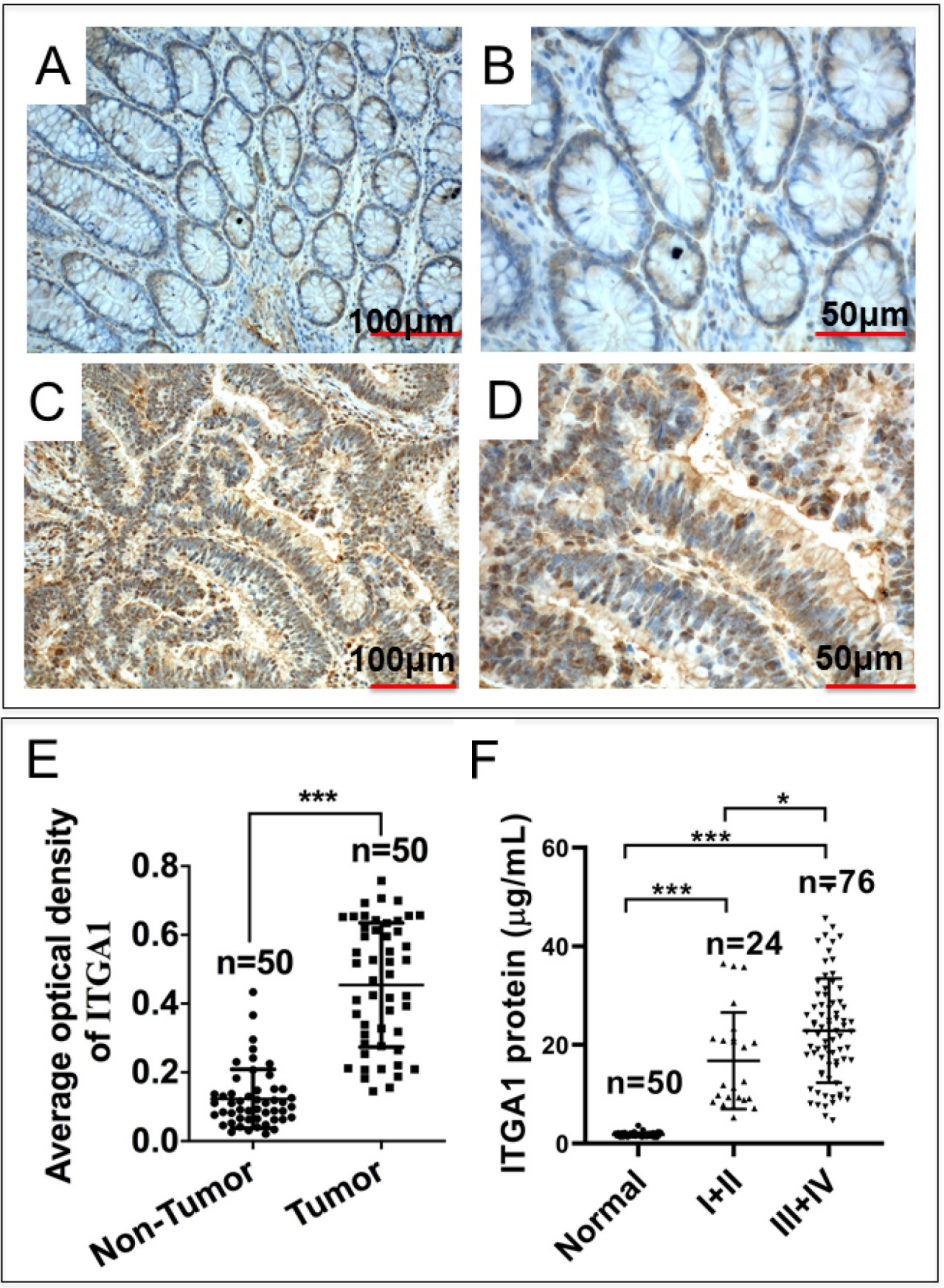
Immunohistochemistry (IHC) staining of ITGA1 protein in human colorectal cancer (CRC) tissues. A negative or weakly positive ITGA1 expression was observed in non-CRC tumor adjacent tissues (A). B, the higher magnification of image (A). C, IHC staining of ITGA1 was visibly expressed in CRC tumor tissues. D, a higher magnification of (C). E, the expression of ITGA1 in non-CRC and CRC tumor determined by the average optical density (AOD) values of IHC. F, the serum concentration of ITGA1 in healthy subjects and CRC patients (TNM stage I+II and III+IV). More abundant ITGA1 protein was detected in the sera of the CRC patients, especially patients in the advanced (III+IV) stage, compare to healthy subjects and non-CRC tumor adjacent tissues, *p<0.05, ***p<0.001. Bars in A and C: 100 μm; Bars in B and D: 50 μm.

Intriguingly, our mRNA expression profiles of human colorectal tissues and precancerous detecting with the Affymetrix Human Genome U133 Plus 2.0 Array shown that the abundance of *ITGA1* transcript was dramatically increased in tumor tissues compared to the paired adjacent normal non-tumor tissues of CRC patients (p = 1.95E-17). In order to investigate the biological significance of ITGA1 in CRC, we initially evaluated the ITGA1 expression at protein level by immunohistochemical (IHC) staining in 50 pairs of CRC tissues and the matched adjacent non-cancerous tissues (Fig. 1A-E). IHC results showed that ITGA1 protein was aberrantly expressed in CRC tumor tissues (Fig. 1A, 1B), but not in matched adjacent non-cancerous tissues (Fig. 1C, 1D). In addition, the average optical density (AOD) values of ITGA1 IHC showed that the overall ITGA1 protein in CRC tumor tissue was more abundant than their matched adjacent non-cancerous tissues (p<0.001) (Fig. 1E).

Furthermore, in order to determine whether ITGA1 is involved in CRC progression, serum concentration of ITGA1 was evaluated in CRC patients and healthy subjects. Serum concentration of ITGA1 protein was 1.79 ± 0.43 μg/mL for healthy subjects and 4.19 ± 1.82 μg/mL in CRC patients (Table 1). Our result showed that ITGA1 expression was elevated in CRC patients compared to healthy subjects (p<0.001). Interestingly, the relevant analysis of serum concentration of ITGA1 protein and clinical pathological assessment further revealed a positive correlation of ITGA1 with the metastatic TNM stages of CRC (p<0.001) (Fig 1F, Table 1). Of note, no correlation was determined among serum concentration of ITGA1 with the gender, age, Duke's staging (A, B, C, D), colorectal cancer biomarkers CEA, CA125 (p>0.05) (Table 1). However, a positive correlation of ITAG1 protein with circulating CA199 of CRC patients was detected (p<0.05) (Table 1). These data suggest that a strong correlation between the ITGA1 protein and CRC clinicopathological feature and pathogenesis, implying that ITGA1 may play a role in the CRC progression.

### An increased expression of ITGA1 is associated with CRC metastasis

**Figure 2:**
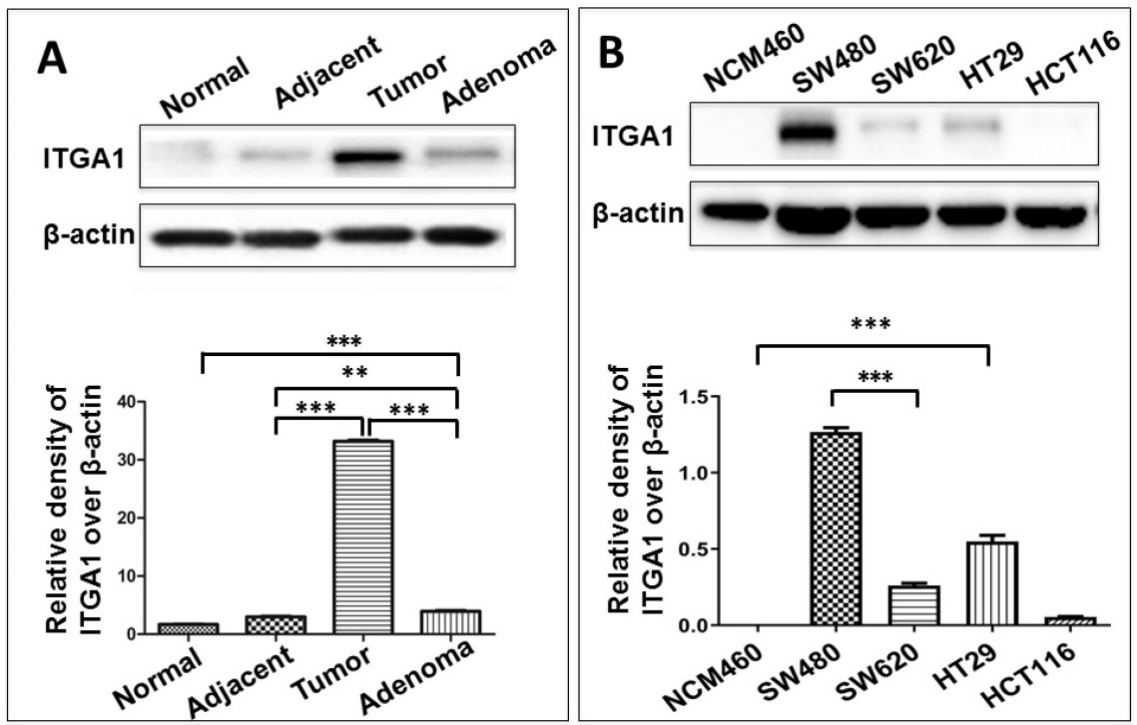
Immunoblotting analysis of ITGA1 expression in colorectal cancer (CRC) tissues and CRC cells. A, representative immunoblotting of ITGA1 protein in CRC tissues (top panel). More abundant ITGA1 protein was detected in CRC tissue compared to normal tissue and the highest ITGA1 protein was detected in the metastatic CRC tumor tissues. B, representative blots of ITGA1 expression in four human CRC cell lines (SW480, SW620, HT29, HCT116) and one normal colonic epithelial cell line (NCM460) (top panel), and a densitometric analysis the relative levels of ITGA1 protein (bottom panel). Error bars represent mean ± SD. Statistical analysis was performed using Student's *t*-test. Compared to normal cell lines or normal tissues, **p<0.01 and ***p<0.001 (n=3).

To study ITGA1 in CRC, we compared ITGA1 protein expression across multiple colorectal tissue types and cell lines (Fig. 2). CRC tumors expressed more abundant ITGA1 protein than non-tumor tissues adjacent to resected tumors, and adenoma samples (Fig. 2). Interestingly, semi-quantitative analysis of ITGA1 protein was even higher in tumor than adenoma (p<0.01) (Fig. 2A, bottom panel). On the other hand, we also found that more abundant ITGA1 protein in colon cancer cell lines (SW480, SW620, HT29) compared to normal colonic epithelial NCM460 cells. The ITGA1 expression was related to the progressive property of CRC cells, i.e. more abundant ITGA1 was observed in highly metastatic SW480 cells than in the relatively less metastatic HCT116 cells (Fig. 2). These results suggest that ITGA1 may relate to the metastasis of CRC.

### Impacts of ITGA1 on the progressive characteristics of CRC cells *in vitro*

**Figure 3:**
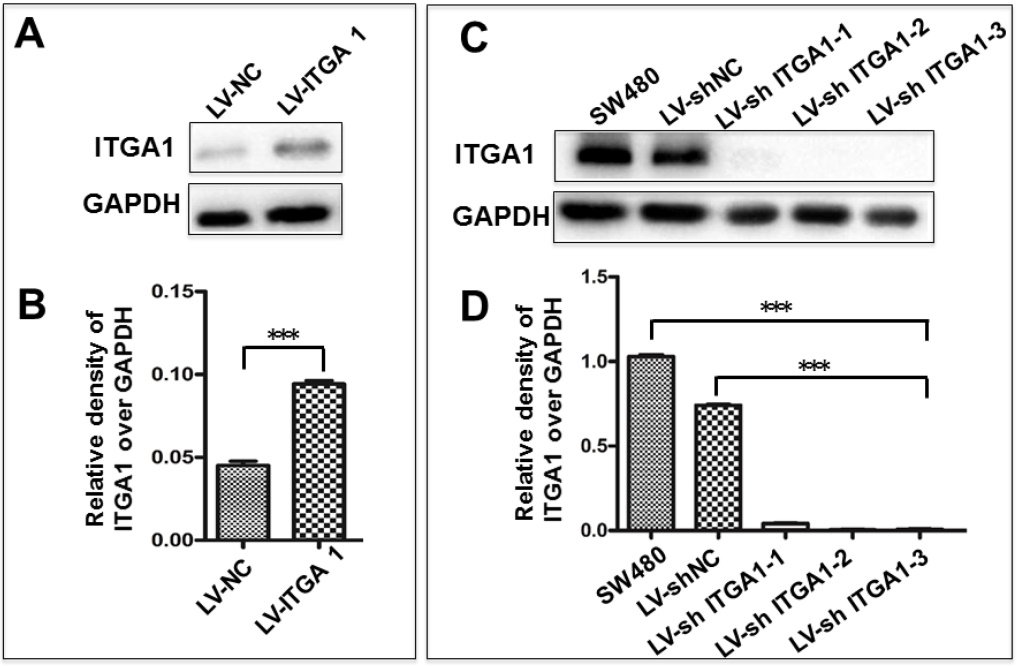
Generation of colorectal cancer cells overexpressing or silencing ITGA1**.** Indicated CRC SW480 cells and normal colon epithelial NCM460 cells were infected with LV-shNC, Lv-shITGA1 and LV-ITGA1, Lv-NC, respectively. A, immunoblotting analysis of ITGA1 expression in NCM460 infected with lentiviral vectors showed that ITGA1 was significant higher expressed in NCM460 compared to Lv-NC cells. B, semi-quantitative analysis of the relative levels of ITGA1 proteins in (A) by a densitometric analysis. Compared to LV-NC infected cells, ***p<0.001. C, immunoblotting analysis of ITGA1 expression in SW480 infected with Lv-shITGA1 showed that ITGA1 was significant inhibited in SW480 compared to LV-shNC cells. D, semi-quantitative analysis of the relative levels of ITGA1 proteins in (C) by a densitometric analysis. Error bars represent mean ± SD. Statistical analysis was performed using Student's *t*-test. Compared to Lv-sh-NC infected cells, ***p<0.001 (n=3).

**Figure 4:**
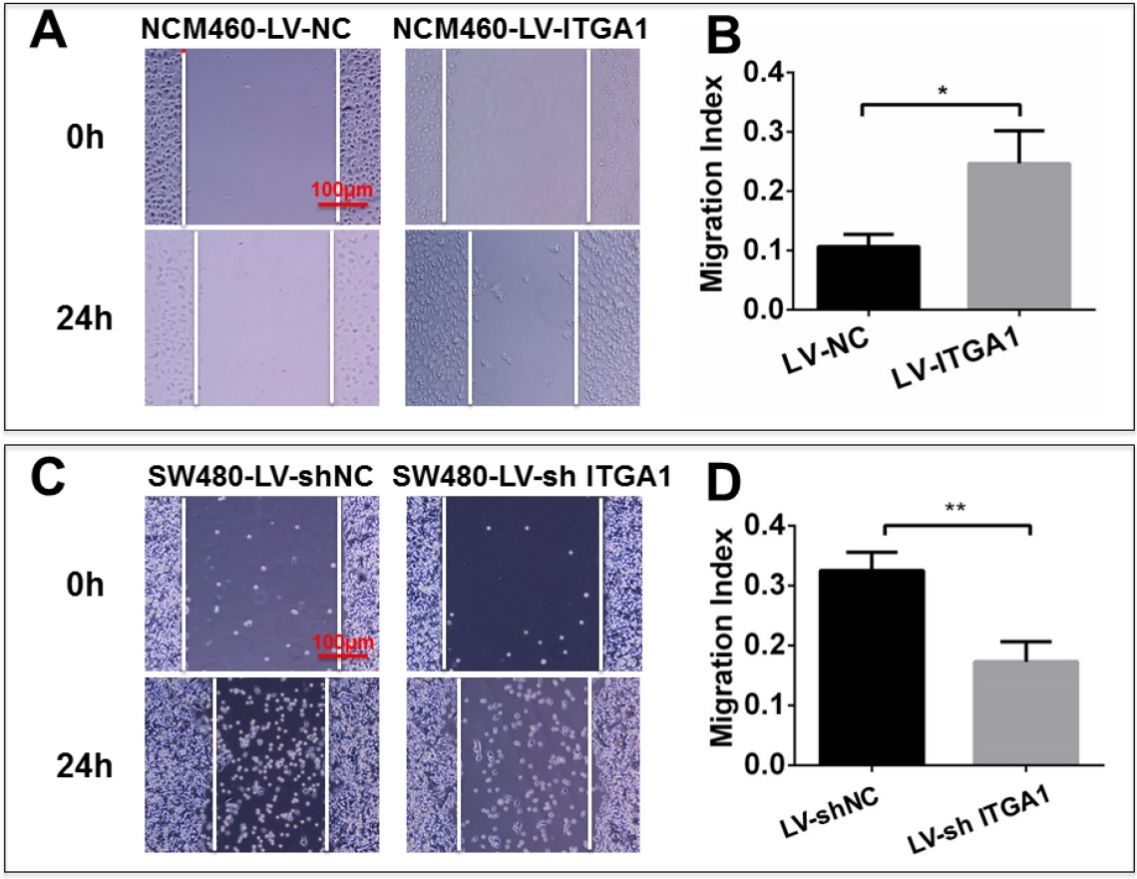
ITGA1 promotes cell migration in colorectal cancer (CRC) cells *in vitro* determined by scratch assay. The SW480 and NCM460 were infected with LV-shNC, LV-shITGA1 and LV-ITGA1, LV-NC, respectively. The capability of cell migration was accessed by cell scratch wound healing assay. A and C, representative images of scratch assay of NCM460, SW480 cells infected with indicated viruses, respectively. B and D, relevant quantification of the results of cell migration index (A) and (C), respectively. ITGA1 showed an ability to promote CRC cell migration. Error bars represent mean ± SD. Statistical analysis was performed using Student's *t*-test. Compared to Lv-NC or LV-shNC infected cells, * and ** represent p<0.05 and p<0.01, respectively (n=3).

**Figure 5:**
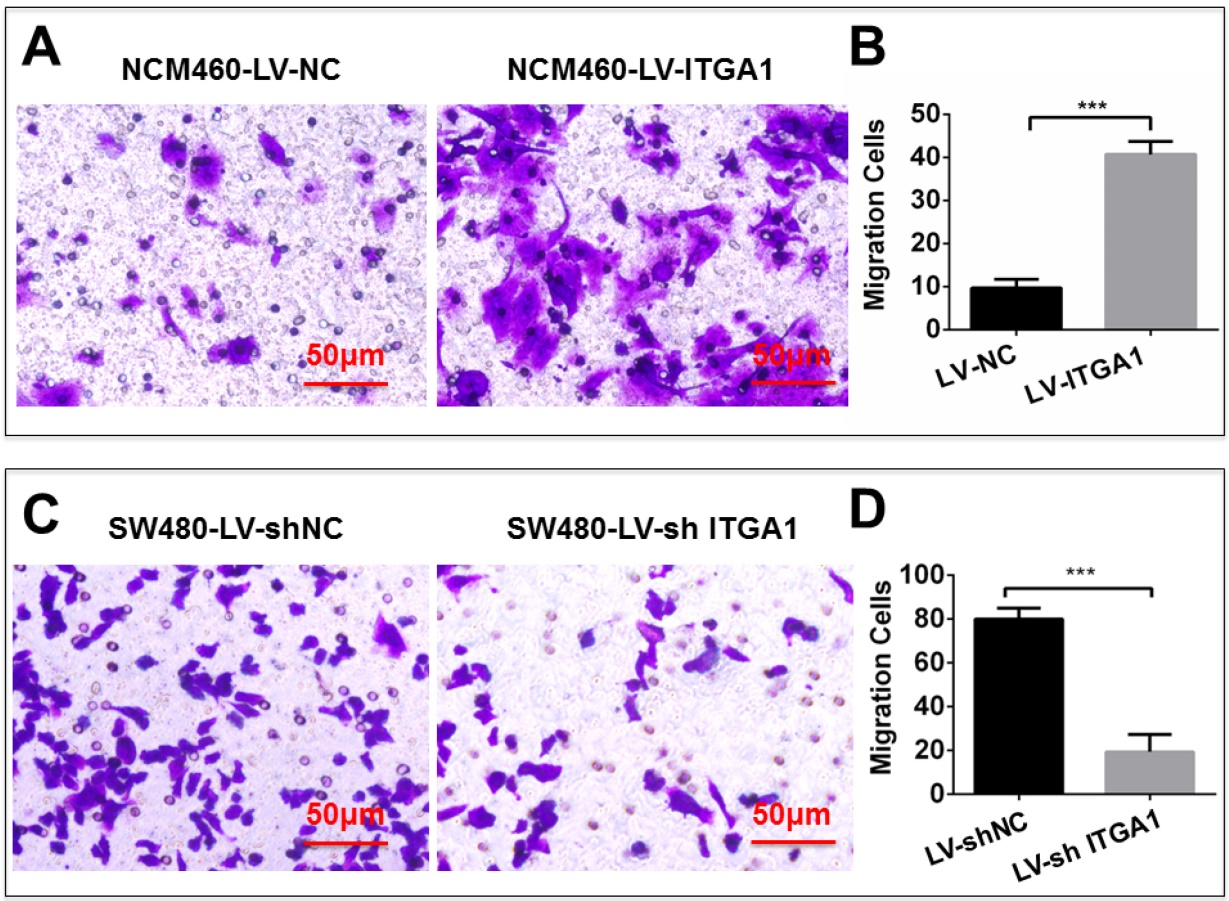
ITGA1 promotes cell invasion in colorectal cancer cells *in vitro* determined by transwell matrigel invasion assay. NCM460 and SW480 cells were infected with LV-ITGA1, LV-NC, and LV-shNC, LV-shITGA1 respectively before they were used for invasion analysis by transwell matrigel invasion assay. A and C, representative images of invasion assays of NCM460 and SW480 cells infected with indicated viruses. B and D, relevant quantification of the numbers of invasive cells. Error bars represent mean ± s.d. Statistical analysis was performed using Student's *t*-test. Compared to Lv-sh-NC infected cells, ***p<0.001 (n=3).

Based on aforementioned results, SW480 and NCM460 cell lines were thus selected for further testing in this study. They were representative cells of a relatively high and low endogenous expression of ITGA1, respectively. The potential impacts of ITGA1 on the progressive properties of CRC cell lines were then explored in SW480 and NCM460 cells by overexpression or knockdown of ITGA1 using lentiviral vector-mediated gene transduction. An overexpressed ITGA1 protein was found in the LV-ITGA1-infected NCM460 cells (Fig. 3A). The cell migration (Fig. 4A and 4B) (p<0.05) and invasion (Fig. 5) (p<0.001) were significantly increased with LV-ITGA1-infected cells compared to LV-NC-infected cells, as determined by scratched wound healing assay (Fig. 4A and 4B) and Transwell invasion assay (Fig. 5A and 5B). Conversely, four ITGA1 shRNAs showed capacities to knockdown endogenous ITGA1 protein expression in SW480 cells, the cell line with more progressive properties (Fig. 3A). The shRNA-mediated silence of *ITGA1* gene led a significant reduction of both cell migration in scratched wound healing assay (Fig. 4C and 4D) (p<0.01) and cell invasion on Transwell invasion assay (Fig. 5C and 5D) (p<0.001), as compared with the LV-shNC-infected group. These results suggest that ITGA1 enhances the progressive potency of CRC cells in terms of cell migration and invasion.

### ITGA1 activates the Ras/Erk signaling pathway

**Figure 6:**
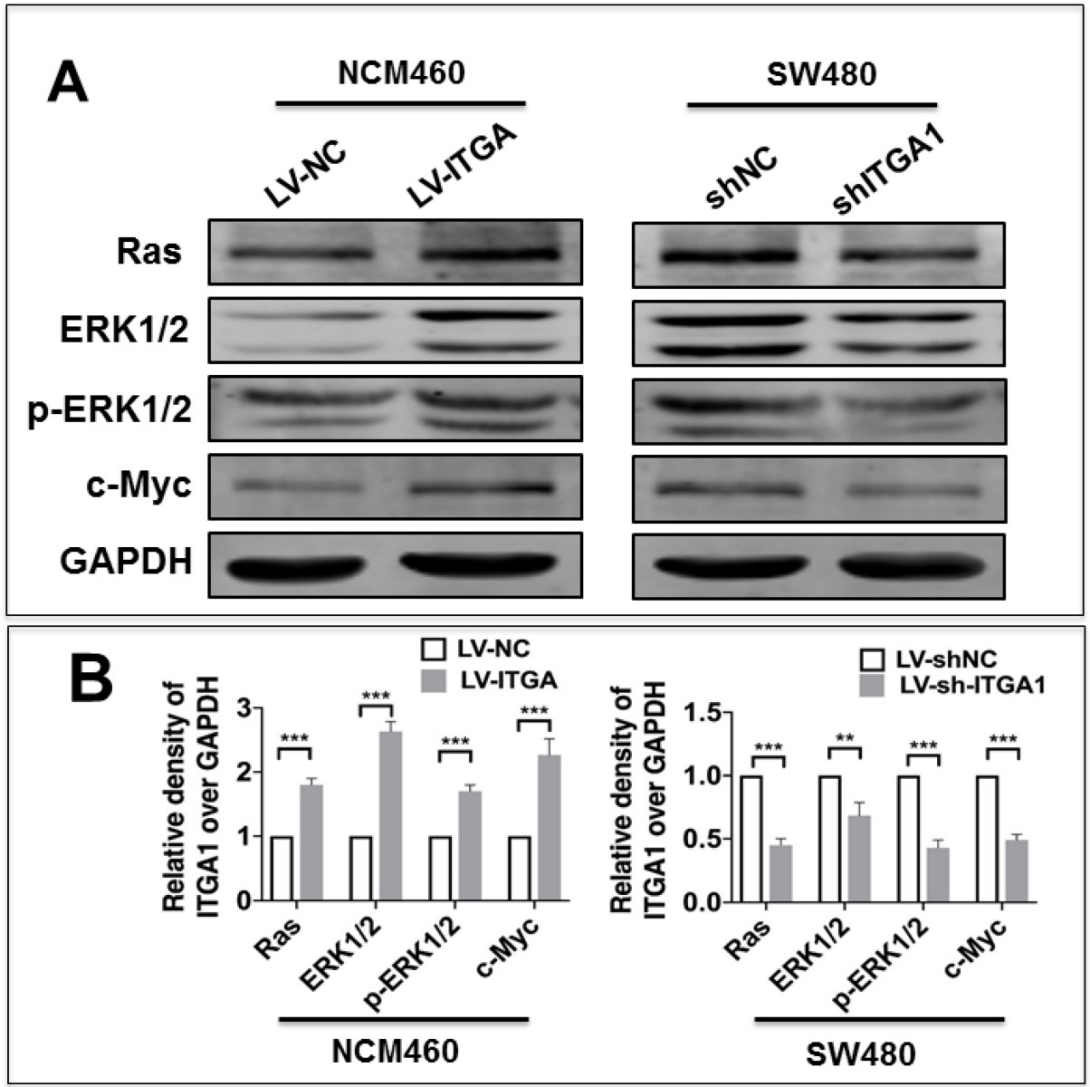
Immunoblotting analyze the Ras/ERK pathway signaling pathway related protein expression. The SW480 and NCM460 were infected with LV-shNC, LV-shITGA1 and LV-ITGA1, LV-NC, respectively. (A) Immunoblotting assay determined the expression of Ras, ERK1/2, p-ERK1/2 and c-Myc. (B) The relative levels of proteins detected as evaluated by a densitometric analysis of interest proteins in (A). Data represented the mean ± SD from three independent triplicated experiments (n=3). Compared with LV-NC or LV-shNC ***p<0.001.

Integrin has been shown to affect tumorigenesis via the Ras/Erk pathway 19. However, whether ITGA1 affects tumorigenesis of CRC via the Ras/Erk pathway requires further to investigate. In order to elucidate the mechanism by which ITGA1 promoted the progressive characteristics of CRC *in vitro*, the relative expression of proteins in the Ras/Erk signaling pathway was evaluated. First, we found that stably overexpressing ITGA1 using LV-ITGA in low endogenous expression NCM460 cells significantly increased Ras, ERK1/2, p-ERK1/2 and c-Myc expression (p<0.001), but stably knocking down ITGA1 in high-metastasis SW480 cells with LV-shITGA1 significantly diminished Ras, ERK1/2, p-ERK1/2 and c-Myc expression (Fig. 6) (p<0.001). These results indicated an involvement of Ras/Erk signaling in ITGA1-mediated progressive capacity of CRC cells.

### ITGA1 enhances the tumorigenesis of CRC cells *in vivo*

**Figure 7:**
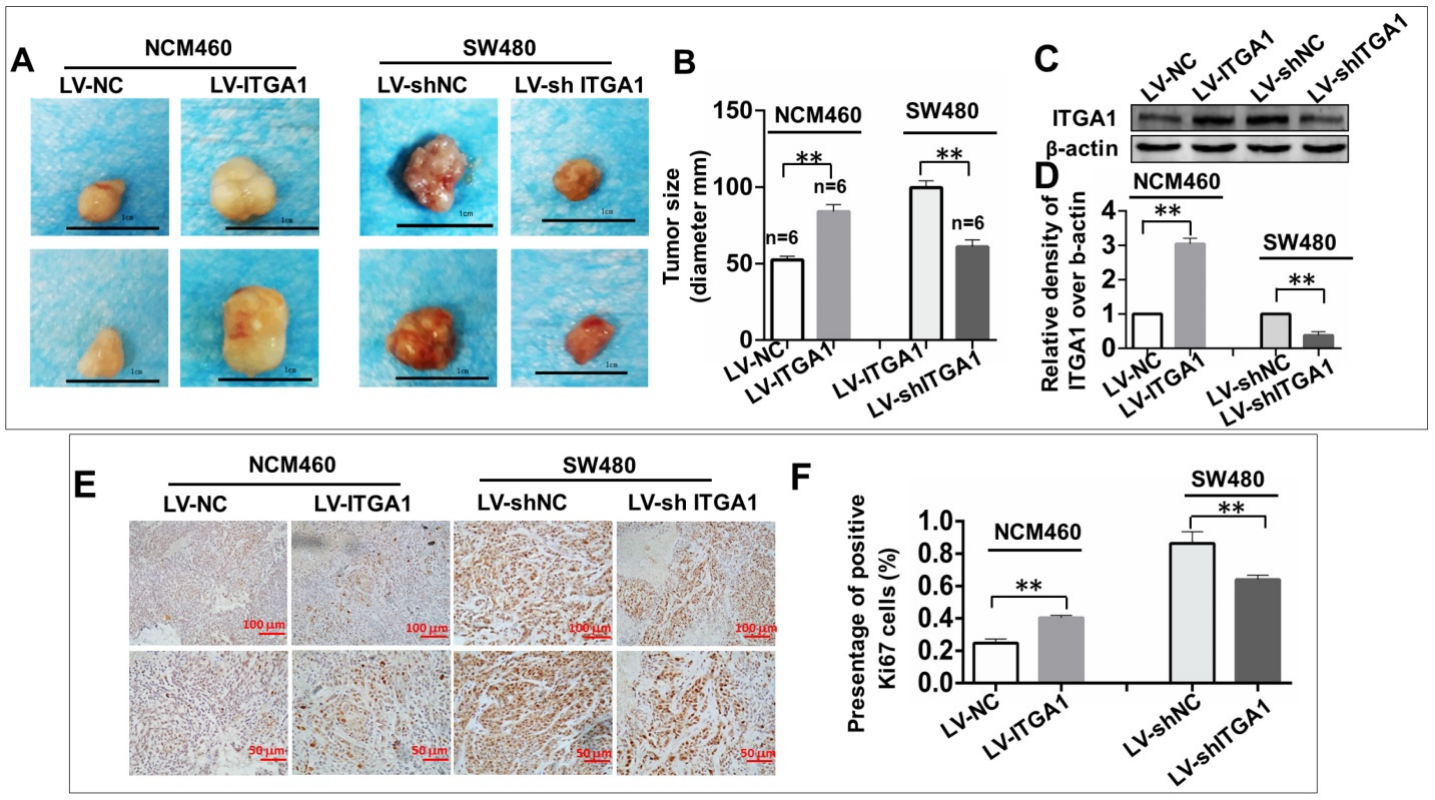
ITGA1 promotes the tumorigenicity in colorectal cancer (CRC) cells *in vivo*. 6-8 week-old female nude mice were subcutaneously injected with 200 μL Matrigel (BD) containing 2×10^6^ virus-infected SW480 or NCM460 cells. The formation of tumor was evaluated at 3 weeks following the cell transplantation. A, images showed the sizes of tumors formed from NCM460 cells infected with LV-ITGA1 and LV-NC (left panel), and SW480 cells infected with LV-shITGA1 and LV-sh-NC (right panel). B, relevant quantification of the sizes (diameter) of tumors. These results showed that an ability of ITGA1 promotes CRC cell tumorigenicity. C, images showed Western blot of ITGA1 expression in the tumor tissues of nude mice. D, The relative levels of ITGA1 detected as evaluated by the relative density over β-actin in (C). E, representative images of Ki67 expression in the tumor tissues by immunohistochemistry. F, relevant quantification of the numbers of Ki67 positive cell. Compared with LV-NC or LV-sh-NC group, ** and *** represent p<0.01 and p <0.001, respectively. Data represented the mean ± SD from three independent triplicated experiments (n=6). Bars in A: 1.0 cm; Bars in E: 100 μm (up panel) and 50 μm (bottom panel), respectively.

To investigate the impact of ITGA1 in the tumorigenicity of CRC cells *in vivo*, an equal number of NCM460 cells with LV-ITGA1 and SW480 cells with LV-shITGA1 were injected subcutaneously into nude mice and employed for evaluating the oncogenic role of ITGA1 in CRC in a xenograph tumorigenic model *in vivo* (Fig. 7). After tumor excision, tumor size was used to evaluate tumorigenic analysis (Fig. 7). Interestingly, the sizes of tumor derived from normal colonic epithelial NCM460 cells with overexpressing ITGA1 were significantly increased compare to the cells infected with LV-NC control group (p<0.01) (left panel in Fig. 7A and 7B). Conversely, a knockdown of ITGA1 in highly metastatic CRC SW480 cells significantly inhibited its tumorigenicity as compared to the control group (p<0.01) (right panel in Fig. 7A, and 7B). For both LV-ITGA1 NCM468 and LV-shITGA1 SW480 derived tumors, the regulation of ITGA1 was validated using Western blot (Fig 7C and 7D). These results showed that downregulated of ITGA1 could inhibit tumor size. Furthermore, from the proliferation marker Ki67, we further revealed that more abundant Ki67-positive proliferative cells were found in tumors derived from NCM460 cells infected with LV-ITGA1 compared to cells infected with LV-NC (p<0.01) (Fig. 7E and 7F). Consistently, the shRNA-mediated silencing of *ITGA1* in SW480 cells led a significant decreased number of Ki67-positive cells in tumor xenograph tissue relative to the control cells (p<0.01) (Fig. 7E and 7F. Taken together, these results suggest that ITGA1 plays an important role in regulating progressive properties and tumorigenesis of CRC cells.

## Discussion

Increasing evidences have suggested that integrins play crucial roles in the development and progression of human cancers by activating different signaling pathways 20-22. ITGA1 belongs to one of integrin, which involves in cell adhesion to the extracellular matrix (ECM), growth, proliferation, migration, survival and apoptosis by regulating inside-out and outside-in signaling 23. In this regard, ITGA1 was found to mediate signals in promoting cancer growth of lung cancer cells in the mice model 24, and blocking ITGA1 could reduce cell invasion and adhesion in a mice breast cancer model 25. Therefore, ITGA1 has been recognized as a novel potential prognostic marker and therapeutic target for human cancers, including metastatic melanoma 26, lung cancer 24, hepatocellular carcinoma (HCC) 22.

It has been reported that ITGA1 was expressed abnormally in colorectal tumors and was elevated in colorectal tumor cells 12, 14. But whether the abundance of ITGA1 in serum and tissue could be used as early diagnosis and treatment of a tumor marker has not yet reported. Indeed, ITGA1 protein was up-regulated in 65% of colorectal cancers 14. Some studies have shown that the expression of ITGA1 was up-regulated in human CRC tissues and cell lines 12, 21 and played an active role in CRC progression 12, 14.

Moreover, we also examined the concentration of ITGA1 in the sera of CRC patients and analyzed the correlation with clinicopathological features, stages and colorectal neoplasms markers of colorectal cancer. The results showed that significantly more abundant of ITGA1 protein was detected in CRC patients compared to heathy group (p<0.001). Even more notably, a significantly higher level of serum ITGA1 was a positive correlation with circulating CA199 of CRC and clinicopathological feature and pathogenesis. Take together, our findings reveal the elevated ITGA1 expression levels in metastatic CRC tumor tissues and sera suggest that the clinical relevance of ITGA1 in CRC metastasis.

The identification of ITGA1 was involved in cell invasion and metastasis in hepatocellular carcinomas 27. In our study, we demonstrated that ITGA1 was highly expressed in CRC tumors. Importantly, the increased expression of ITGA1 in NCM460 cell showed an ability to promote cell migration, invasion *in vitro* and enhance proliferation. The RAS/MEK/ERK signaling pathway has been reported to associate with the CRC and have been studied due to involvement in the regulation of cell proliferation, survival and tumor invasiveness 28, 29. An early study demonstrated that ITGA1 could recruit caveolin-1 and promote Src and the growth factor receptor-bound protein 2 (Grb 2), resulting in activating RAS/MEK/ERK signaling pathway in CRC cells. Actually the RAS/MEK/ERK signaling pathway could be activated by many tumor-related proteins, including α2β1 integrin 30. In this study, we found that overexpression of ITGAI1 significantly activated ERK1/2 as a key component in the Ras/Erk pathway in CRC migration and invasion. The results were supported by some previous studies. It was reported that integrin α1β1 could regulate the activation of focal adhesion kinase (FAK)/Src and p130Cas/c-Jun signaling pathway to promote colon cancer cell invasion 15. Emerging evidences have shown that ITGA1 promoted proliferation of CRC cells by regulating the Ras/Erk pathway 12, and c-Myc was also involved in the regulation of ITGA1 expression in CRC cells 21. Together with ours, these data suggest that ITGA1 may be a key molecule to engage the cancer cell progression.

For CRC, both female and male mice animal model are used to study integrins related cancer research 31. Recently, in experiments performed by Ju et al found that hypoxia induces the expression of integrins, which bind to collagen including ITGA1, to promote tumor metastasis in female NOD-SCID or BALB/c mice (age of 5-7 week) 32, thus regarding there is no strong sex-linked in the CRC. We adopt female mice for our study. we were able to show, using lentivirus transfected overexpressing or silencing ITGA1 in CRC cells, that the overexpressing ITGA1 CRC cells could promote tumorigenesis. Further immunostaing for specific proliferation marker Ki67 shows that more abundant Ki67-positive proliferative cells were expressed in tumors derived from overexpressing ITGA1 CRC cells (Fig. 7). These findings were in line with some other cancers. For instance, ITGA1 was upregulated in pancreatic intraepithelial neoplasms and pancreatic ductal adenocarcinoma (PDAC) tissues, and was able to mediate PDAC cell migration 33. Indeed, knockout ITGA1 could inhibit Kras-induced lung cancer* in vivo*, including decreased incidence of primary lung tumors and long survival by reducing cell proliferation and angiogenesis 24.

## Conclusion

In the present study, our results showed that ITGA1 was up-regulated in tumor tissue and serum of CRC patients, which was significantly associated with the metastatic TNM stages of CRC and circulating CA199. We also found that ITGA1 plays an important role in regulating the biological behaviour of CRC cells, including migration, invasion and tumorigenicity. Besides, we found that ITGA1 could activate RAS/MEK/ERK signaling pathway cascades and led to activation of Ras and ERK1/2 in CRC cells, which in turn activated c-Myc, subsequently promoted tumor cells proliferation, migration, invasion and tumorigenicity. This study therefore suggests that the ITGA1 may play an important role in pathogenesis of CRC, which may act as a potential biomarker in the diagnosis and treatment of CRC.

## Figures and Tables

**Table 1:** Correlation of ITGA1 with clinicopathological features, stages and colorectal neoplasms markers of colorectal cancer

Group	Number of cases	Concentration μg/mL	t/F value	p value
**Serum**			39.3/5.93	
Healthy subjects	50	1.79±0.43		<0.0001
CRC patients	100	4.19±1.82		
**Gender**			1.072/1.363	
Male	27	4.56±1.99		0.177
Female	73	3.60±1.45		
**Age**			0.634/-	
40-49	20	4.73±2.44		0.596
50-59	33	4.30±1.70		
60-69	22	4.49±1.74		
70-	25	3.47±1.55		
**Diagnosis**			1.849/0.903	
Rectal cancer	40	3.80±1.38		0.370
Colon cancer	60	4.41±2.18		
**TNM staging**			5.56/-	
I + II	24	2.82±0.81		<0.0001
III + IV	76	4.71±1.91		
**Duke's staging**			2.5/-	
A	16	3.08±0.81		0.067
B	19	4.03±1.93		
C	22	3.27±1.19		
D	43	5.19±2.14		
**CEA**			1.765/1.251	
Positive	66	4.50±2.04		0.215
Negative	34	3.60±1.25		
**CA125**			1.215/0.155	
Positive	56	3.10±1.27		0.877
Negative	44	4.14±1.59		
**CA199**			4.772/2.339	
Positive	67	4.76±2.05		0.022
Negative	33	3.10±0.97		
**Tumor metastasis**			1.062/0.980	
Yes	55	4.49±2.02		0.331
No	45	3.80±1.42		

Note: Data represented the mean ± SD analyzed by Student's *t*-test using SPSS. CEA, carcinoembryonic antigen; CA199, carbohydrate antigen 199; CA125, carbohydrate antigen 125; t/F value, the statistical values of the F-test and *t*-test, respectively.

## Abbreviations

CA125: carbohydrate antigen 125
CA199: carbohydrate antigen 199
CEA: carcinoembryonic antigen
CRC: colorectal cancer
ECM: extracellular matrix
FAK: focal adhesion kinase
HCC: hepatocellular carcinoma
IHC: immunohistochemistry
ITGA1: integrin α1
LV: Lentivirus
PDAC: Pancreatic ductal adenocarcinoma
t-CEA: tissue carcinoembryonic antigen
s-CEA: serum carcinoembryonic antigen
